# Clinical performance of fecal calprotectin, lactoferrin, and hemoglobin for evaluating the disease activity of IBD and detecting colorectal tumors

**DOI:** 10.1002/jgh3.13077

**Published:** 2024-06-04

**Authors:** Tsukasa Yamakawa, Takakazu Miyake, Yoshihiro Yokoyama, Tomoe Kazama, Yuki Hayashi, Daisuke Hirayama, Shinji Yoshii, Hiro‐o Yamano, Satoshi Takahashi, Hiroshi Nakase

**Affiliations:** ^1^ Department of Gastroenterology and Hepatology Sapporo Medical University School of Medicine Sapporo Japan; ^2^ Department of Infection Control and Laboratory Medicine Sapporo Medical University School of Medicine Sapporo Japan

**Keywords:** calprotectin, colorectal tumor, hemoglobin, IBD, Lactoferrin

## Abstract

**Background and Aim:**

Recently, noninvasive fecal markers have been used as indicators of intestinal inflammation in patients with inflammatory bowel disease (IBD). We conducted a clinical validation study to measure fecal calprotectin (Cp), lactoferrin (Lf), and hemoglobin (Hb) levels using an all‐in‐one kit in patients with IBD and colorectal tumors and aimed to clarify the utility of these fecal markers.

**Methods:**

In this study, 104 patients were analyzed, including 25 patients with ulcerative colitis (UC), 20 with Crohn's disease (CD), 48 with colorectal tumors, and 13 healthy controls (HC). Of the 48 patients with colorectal tumors, 14 had invasive cancer. We validated the utility of fecal Cp, Lf, and Hb levels by simultaneously measuring fecal markers in patients with IBD and colorectal tumors.

**Results:**

Fecal Cp and Lf had almost equivalent abilities in detecting clinical remission in patients with UC; however, fecal Cp was slightly superior to Lf. Regarding colorectal tumors, fecal Cp and Lf levels tended to be higher in patients with adenomas and colorectal cancer than in HCs. Although fecal Hb alone had the best sensitivity and specificity for detecting colorectal cancer, it had relatively low sensitivity for detecting advanced neoplasms and colorectal cancer.

**Conclusion:**

Fecal Cp and Lf can be used as almost equivalent biomarkers to assess the clinical activity in patients with UC. Fecal Hb is the most useful marker for screening colorectal cancer; however, adding fecal Cp and Lf may compensate for the low sensitivity of detecting for advanced colorectal tumors based on Hb alone.

## Graphical abstract

This study presented that fecal biomarkers, calprotectin (Cp) and lactoferrin (Lf), can be almost equivalent biomarkers to assess the clinical activity in patients with UC. Moreover, fecal Cp and Lf levels are also elevated in patients with colorectal tumors, although fecal hemoglobin is the most useful marker for detecting colorectal cancer, adding fecal Cp and Lf may compensate for the low sensitivity of screening for colorectal tumors based on hemoglobin alone.

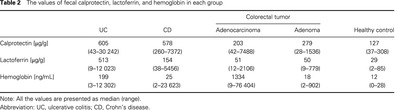

## Introduction

Noninvasive fecal markers have recently been used as indicators of intestinal inflammation in patients with inflammatory bowel disease (IBD).^1^ Calprotectin (Cp), which is released by leukocytes in the inflamed intestinal tract, is a major component of cytoplasmic protein in neutrophils. In clinical practice, Cp is widely used as a fecal biomarker, and outpatients can collect stool samples and submit them for testing on the day of their visit with stability at room temperature for several days.^2^ Lactoferrin (Lf), another fecal marker, is a multifunctional mammalian iron‐binding glycoprotein involved in regulating inflammatory response and maintaining gut homeostasis.^3^ Lf is a component of specialized granules of neutrophils and is a sensitive inflammatory biomarker because it degranulates more readily in response to stimuli than neutrophil elastase.^4^ In previous reports, both fecal Cp and Lf correlated with IBD disease activity.^1^, ^5^, ^6^, ^7^

Although the fecal immunochemical test (FIT) is the best screening method for colorectal cancer, fecal Cp and Lf levels are also associated with the degree of progression in patients with advanced colorectal cancer.^8^, ^9^ However, little is known about the clinical utility of Cp and Lf in colorectal tumors.

This study aimed to validate the utility of fecal Cp, Lf, and hemoglobin (Hb) levels by simultaneously measuring fecal markers in patients with IBD and colorectal tumors.

## Method

### Patient eligibility

For patient recruitment, we excluded those who had undergone total colectomy before and those from whom we could not obtain written informed consent. A total of 120 patients who received colonoscopy at Sapporo Medical University Hospital participated in this study between March 2020 and March 2022, including 29 with ulcerative colitis (UC), 25 with Crohn's disease (CD), 48 with colorectal tumors, and 18 healthy controls (HC). We defined HC as a patient without colorectal adenoma or cancer on colonoscopy and coexisting systemic inflammatory diseases that could affect the fecal markers results.

**Figure 1:**
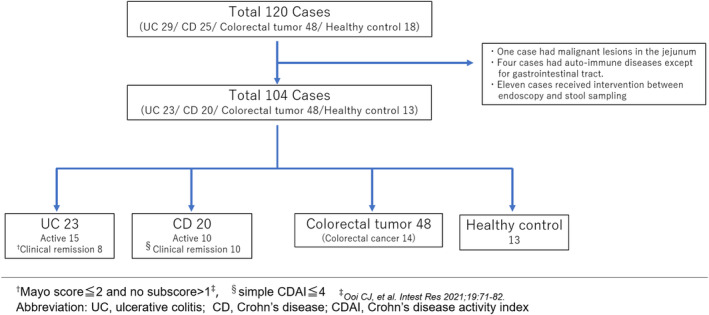
Flowchart. A total of 120 patients participated in this study, including 29 with UC, 25 with CD, 48 with colorectal tumors, and 18 HCs. We excluded 11 patients who received intervention between endoscopy and stool sampling in UC or CD groups, four with comorbidities that caused systemic inflammation, and one HC with jejunal malignancy. Finally, 104 patients were analyzed.

All enrolled patients underwent colonoscopy and stool sampling, and each disease was diagnosed based on clinical, endoscopic, and histopathological findings.^10^ We excluded 11 patients who underwent intervention between endoscopy and stool sampling in the UC and CD groups, four with comorbidities that caused systemic inflammation, and one patient in the HC group with jejunal malignancy. Finally, 104 patients were analyzed (Fig. 1).

### Study design

This prospective, single‐center, observational study was conducted at Sapporo Medical University Hospital. One of the primary endpoints was to validate the clinical performance of fecal Cp, Lf, and Hb in detecting clinical remission in patients with IBD. The other primary endpoint was to determine the cutoff values of these fecal markers for detecting colorectal cancer and advanced neoplasm in non‐IBD patients.

### Ethics statements

This study was approved by the ethics committee of Sapporo Medical University (IRB No. 312‐132) and was conducted in accordance with the Declaration of Helsinki. All the patients provided written informed consent to participate in the study.

### Measurement of fecal markers

All enrolled patients provided a single stool sample within 72 hours of collection. Fecal Cp, Lf, and Hb levels were measured using a Hemotect NS‐Prime (Alphlesa Pharma, Osaka, Japan), which can measure these fecal markers simultaneously with colloidal gold immunochromatographic assay. In this study, the period between endoscopy and stool collection was limited to one year.

### Assessment of clinical and endoscopic severity in IBD

In the UC group, we evaluated clinical activity using a partial Mayo score, and clinical remission was defined as two or fewer Mayo scores (no subscore >1).^11^ In the CD group, clinical activity was evaluated using the simple Crohn's Disease Activity Index (CDAI), and clinical remission was defined as four or fewer points on the simple CDAI. Endoscopists categorized the disease extent based on the Montreal classification (E1/ E2/ E3 for UC and L1/ L2/ L3/ L4 for CD). Endoscopic severity was assessed according to the Mayo endoscopic subscore (MES) and Ulcerative Colitis Endoscopic Index of Severity (UCEIS) for UC and Simple Endoscopic Score in Crohn's Disease (SES‐CD) for CD. We examined the relationship between fecal marker levels and clinical and endoscopic disease activity scores in both UC and CD groups.

### Diagnosis of colorectal tumor

Of the 48 patients with colorectal tumors on colonoscopy, 14 had invasive cancers, which were diagnosed based on the results of the pathological findings after surgical resection. For the patients whose primary tumor had not been resected, the invasion depth was estimated with the results of the endoscopic examination, CT, and MRI.

### Statistical analysis

Statistical analyses were performed using the IBM SPSS Statistics version 25 (IBM Corp., Armonk, NY, USA). For categorical and continuous data, a 2‐group comparison was performed using the Student's *t*‐test, Mann–Whitney *U* test, and Spearman's rank correlation coefficient, as appropriate, to calculate the statistical significance of the demographic and clinical variables according to the manufacturer's instructions. The Kruskal–Wallis test, followed by Tukey's multiple comparison test, was used to compare continuous variables among three or more groups. A value of *P* < 0.05 was considered statistically significant.

## Results

### Baseline characteristics

In this study, 104 patients were analyzed, including 25 with UC, 20 with CD, 48 with colorectal tumors, and 13 HCs (Fig. 1). The baseline characteristics of the enrolled patients are shown in Table 1. Of the 48 patients with colorectal tumors, 14 had invasive cancers, the clinical stage of which was based on the seventh TNM classification (Table S1, Supporting information).

**Table 1:** Patient characteristics in each group

	UC	CD	Colorectal tumor	Healthy control
Number	23	20	48	13
Age	*52 (19–76)	*32 (18–70)	72 (16–83)	65 (48–83)
Sex (male/female)	12/11	15/5	27/21	5/9
BMI [kg/m^2^]	*21.2 (15.4–28.9)	*20.8 (13.9–29.1)	21.8 (14.7–27.7)	22.5 (18.6–36.3)
Comorbidity				
Diabetes mellitus	5	0	9	0
Cardiovascular	3	0	13	0
Chronic kidney disease	1	1	2	1
Severe liver disease	0	0	4	0
Pulmonary disease	3	0	4	1
Cerebrovascular disease	1	1	2	0
Malignancy	1	0	3	1
Anticoagulant	2 (8.7%)	1 (5.0%)	19 (39.6%)	1 (7.7%)
Post ileocolonic resection	0	7 (35.0%)	6 (12.5%)	5 (38.5%)
Hb [g/dl]	13.0 (7.2–15.6)	13.5 (9.8–15.2)	12.4 (7.9–16.2)	12.7 (11.0–16.7)
CRP [mg/dl]	0.08 (0.01–0.59)	0.1 (0–5.5)	0.07 (0–5.85)	0.02 (0–0.29)
LRG [μg/mL]	12.3 (7.7–37.4)	8.9 (7.7–13.1)	‐	‐

* *P* < 0.05 by Student's *t*‐test in comparison to healthy control group.

Note: Continuous data are presented as median (range).

Abbreviation: BMI, body mass index; CD, Crohn's disease; CRP, C‐reactive protein; Hb, hemoglobin; LRG, leucine‐rich α2 glycoprotein; UC, ulcerative colitis.

### Characteristics and disease activity in IBD

Regarding the disease extent defined by the Montreal classification for patients with UC, two had proctitis (E1), six had left‐sided colitis (E2), and 15 had pancolitis (E3). As for the disease extent defined by the Montreal classification for patients with CD, four had ileal involvement (L1), three had colonic involvement (L2), 13 had ileocolic involvement (L3), and none had isolated upper disease (L4). In the UC group, most patients received 5‐ASA, followed by thiopurine, biologics, and systemic steroids, whereas none received tacrolimus (Table S2). The median partial Mayo score was 2 (range, 0–7). Most patients in the CD group received 5‐ASA, followed by biologics, thiopurines, and systemic steroids. The median simple CDAI score was 1.5 (range, 0–9). Regarding endoscopic severity, the median MES and total UCEIS for patients with UC were 1 (0–2) and 3 (0–5), respectively, while that of median SES‐CD for patients with CD was 5 (0–9). Fifteen of 23 patients with UC and 10 of 20 patients with CD were clinically active.

### Fecal calprotectin, lactoferrin, and hemoglobin levels in each group

In this study, more than half of the patients with clinically active IBD (15 UC and 10 CD) underwent colonoscopy two weeks within the stool sampling, and the majority of these 25 IBD patients did colonoscopy within about a month before and after the stool sampling. Fecal Cp levels in patients with UC, CD, invasive colorectal cancers, colorectal adenomas, and HCs were 43–30 242 μg/g (median, 605 μg/g), 260–7232 μg/g (median, 578 μg/g), 42–7488 μg/g (median, 203 μg/g), 28–1536 μg/g (median, 279 μg/g), and 37–308 μg/g (median, 127 μg/g), respectively (Table 2). Fecal Cp levels were significantly higher in patients with UC and CD than in the HCs (*P* < 0.01) (Fig. S1, Supporting information). Fecal Lf levels in patients with UC, CD, invasive colorectal cancers, colorectal adenomas, and HCs were 9–12 023 μg/g (median, 513 μg/g), 38–5456 μg/g (median, 154 μg/g), 12–2106 μg/g (median, 51 μg/g), 9–779 μg/g (median, 50 μg/g), and 2–85 μg/g (median, 29 μg/g), respectively (Table 2). Fecal Lf levels were significantly higher in patients with UC and CD than in those with colorectal adenomas (*p* = 0.03) and in HCs (*p* < 0.01) (Fig. S1). Fecal Hb levels in patients with UC, CD, invasive colorectal cancers, colorectal adenoma, and HCs were 3–12 302 ng/mL (median, 199 ng/mL), 2–23 623 ng/mL (median, 25 ng/mL), 9–76 404 ng/ml (median, 1334 ng/mL), 2–902 ng/mL (median, 18 ng/mL), and 0–28 ng/mL (median, 12 ng/mL), respectively (Table 2). Fecal Hb levels were significantly higher in patients with colorectal cancers than in those with CD (*P* = 0.03), colorectal adenoma (*P* < 0.01), or HCs (*P* < 0.01) (Fig. S1). In addition, fecal Hb levels were significantly higher in patients with UC than in HCs (*P* < 0.01) (Fig. S1).

**Table 2:** The values of fecal calprotectin, lactoferrin, and hemoglobin in each group

	UC	CD	Colorectal tumor		Healthy control
			Adenocarcinoma	Adenoma	
Calprotectin [μg/g]	605 (43–30 242)	578 (260–7372)	203 (42–7488)	279 (28–1536)	127 (37–308)
Lactoferrin [μg/g]	513 (9–12 023)	154 (38–5456)	51 (12–2106)	50 (9–779)	29 (2–85)
Hemoglobin [ng/mL]	199 (3–12 302)	25 (2–23 623)	1334 (9–76 404)	18 (2–902)	12 (0–28)

Note: All the values are presented as median (range).

Abbreviation: UC, ulcerative colitis; CD, Crohn's disease.

### Correlation between fecal markers and clinical assessment

**Figure 2:**
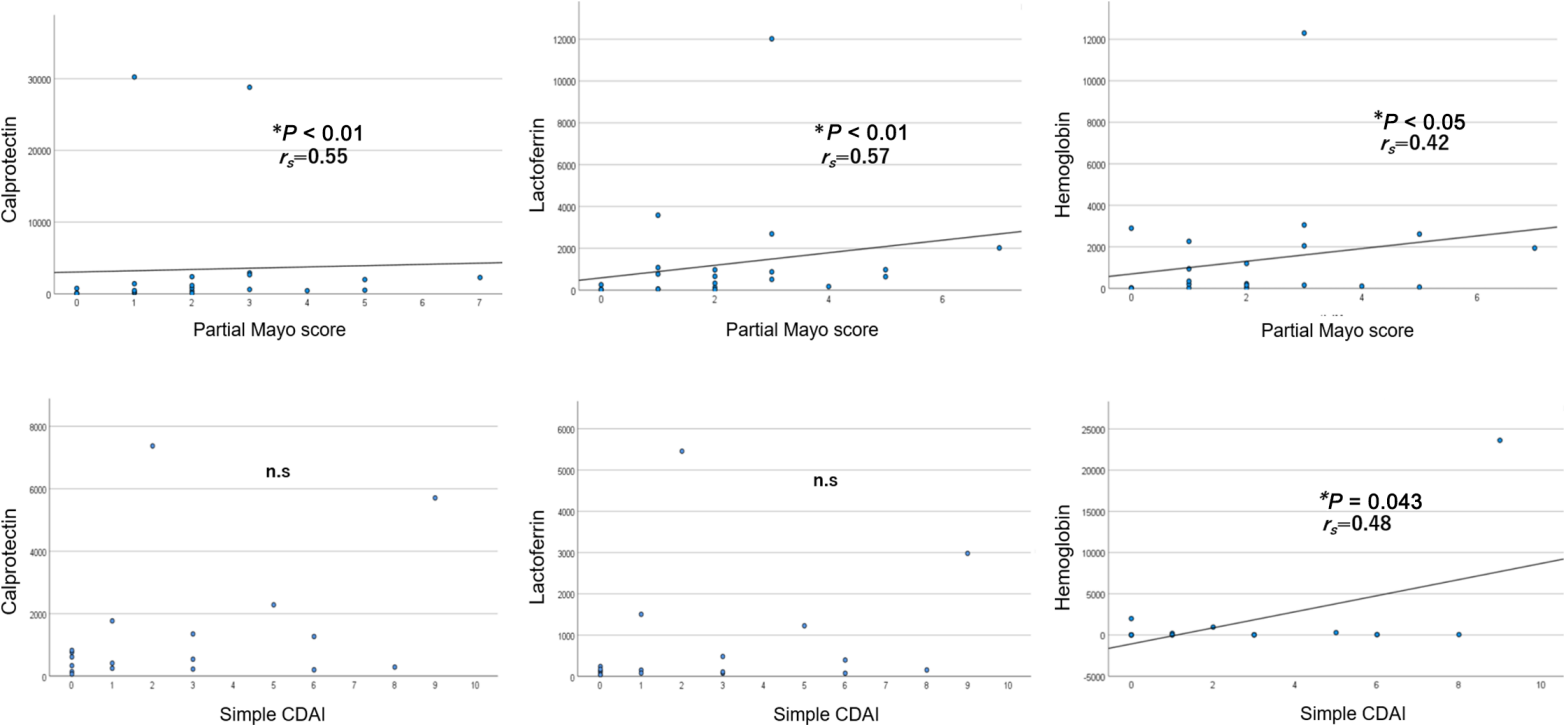
The correlation between fecal markers and clinical activity scores in IBD patients. The association between fecal markers and clinical activity scores. Each vertical axis represents the levels of fecal Cp, Lf, and Hb, and each horizontal axis represents the clinical scores of partial Mayo score for UC and simple CDAI for CD.

We examined the correlation between fecal markers and the partial Mayo score for UC and the simple CDAI score for CD. Partial Mayo score correlated with fecal Cp (*P* < 0.01, *r*_*s*_ = 0.55), Lf (*P* < 0.01, *r*_*s*_ = 0.57), and Hb (*P* < 0.05, *r*_*s*_ = 0.42). The simple CDAI score significantly correlated only with fecal Hb (*P* = 0.043, *r*_*s*_ = 0.48) and showed no correlation with Cp or Lf (Fig. 2).

**Figure 3:**
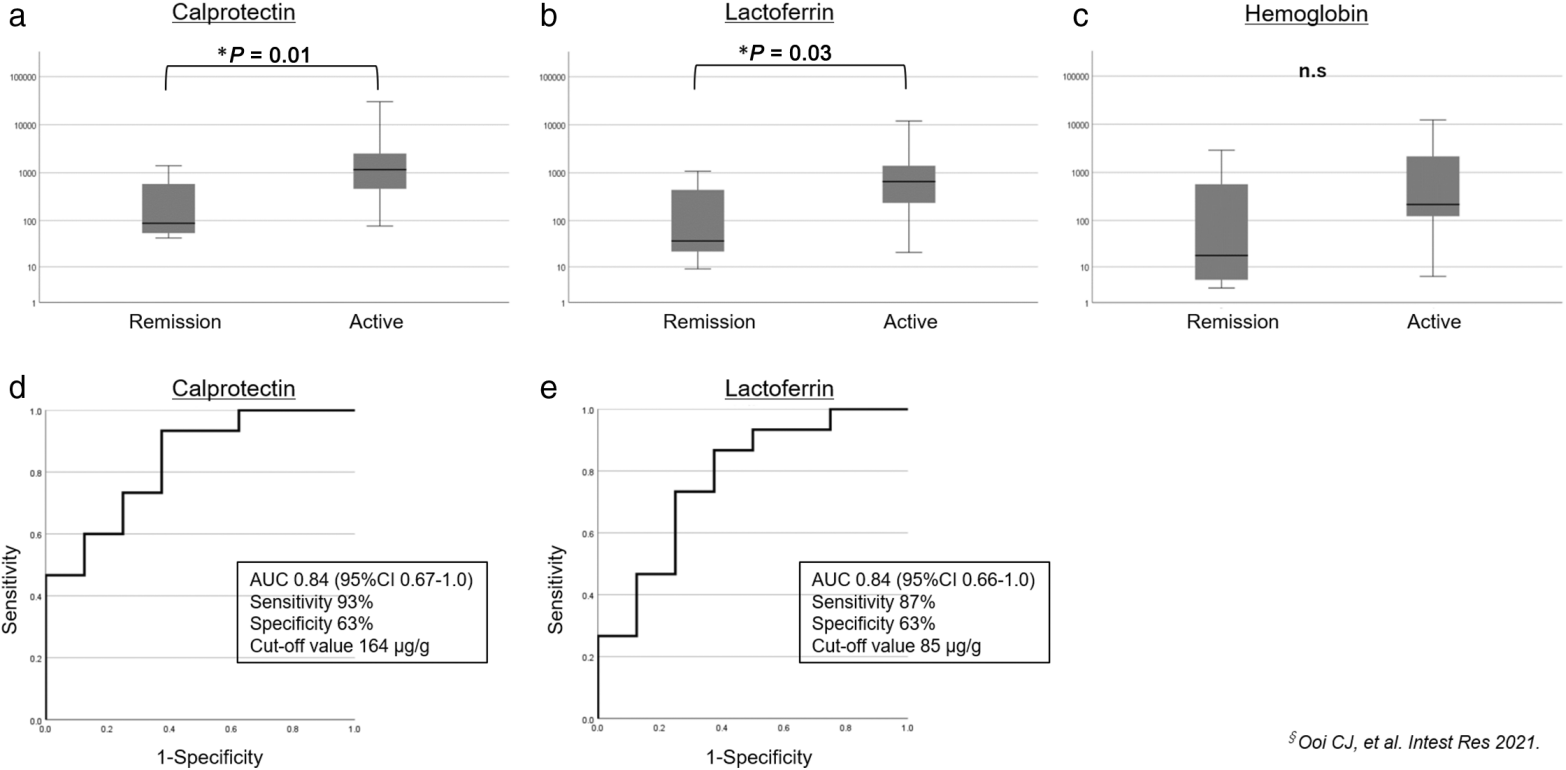
The difference in fecal markers in patients with UC between clinically active and remission. The difference in fecal marker levels in patients with UC in clinically active and remission states. (a) fecal calprotectin, (b) lactoferrin, (c) hemoglobin. The value of the vertical axis is log‐transformed. ROC analysis of (d) fecal Cp and (e) Lf to differentiate active disease from clinical remission^†^ in UC patients. ^†^Mayo score ≤ 2 and no subscore>1 (Ooi CJ, et al. *Intest Res*. 2021;19:71–82).

Regarding disease activity for UC, fecal Cp and Lf were significantly higher in patients with active UC than in those in clinical remission (*P* = 0.01 and *P* = 0.03, respectively); however, there was no significant difference in fecal Hb levels (Fig. 3a–c). Next, we generated a receiver operating characteristic (ROC) curve, and fecal Cp of 164 μg/g (AUC = 0.84, 95% CI 0.67–1.0, sensitivity 93% and specificity 63%) and Lf of 85 μg/g (AUC = 0.84, 95% CI 0.66–1.0, sensitivity 87% and specificity 63%) were the best discriminating levels for predicting active disease in IBD (Fig. 3d,e). However, none of the stool markers showed a significant difference between the active and clinical remission states in the CD group (Fig. S2).

### Correlation between fecal markers and endoscopic severity scores

**Figure 4:**
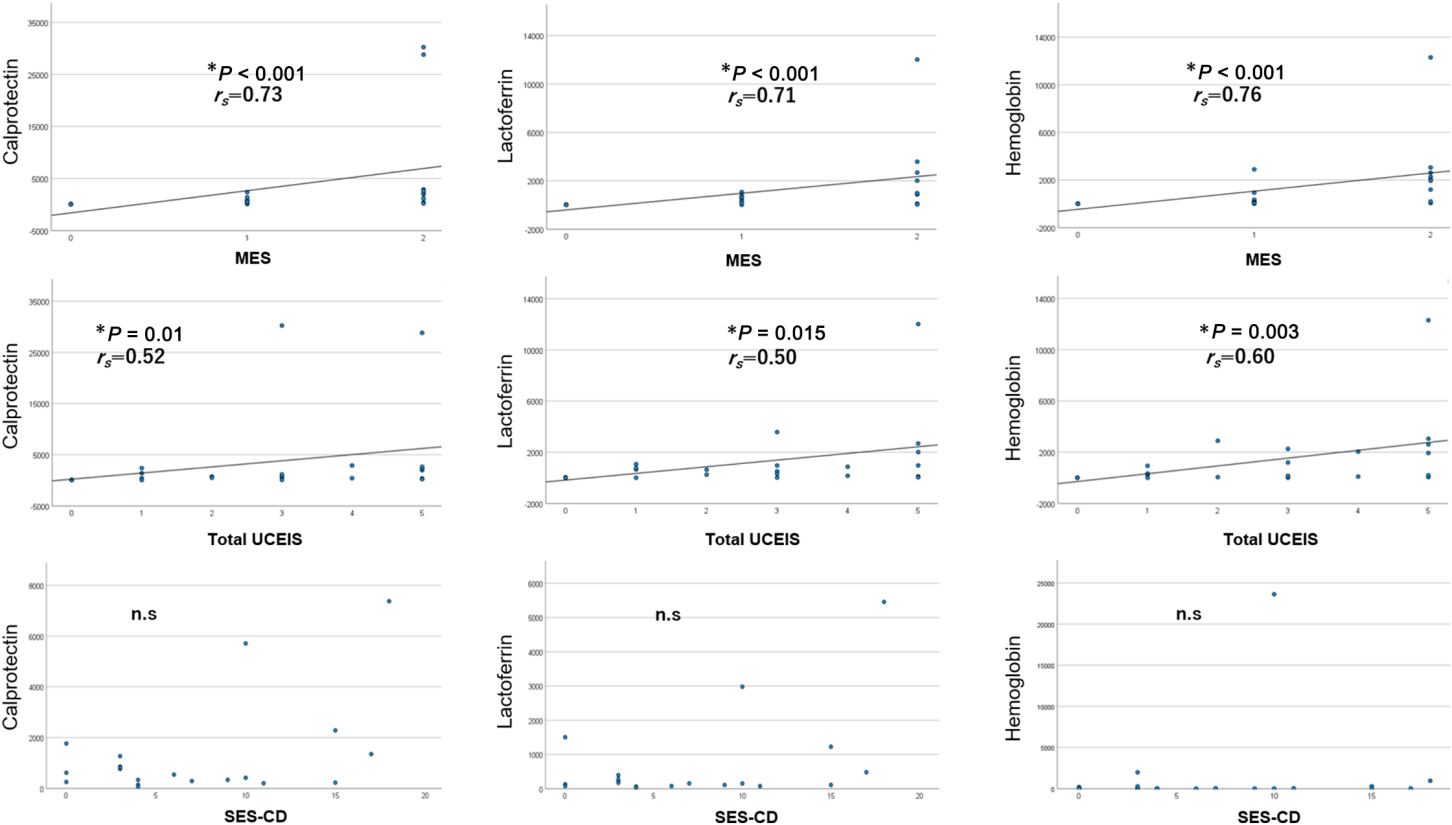
The correlation between fecal markers and endoscopic scores in IBD patients. The association between fecal markers and endoscopic scores. Each vertical axis represents the fecal Cp, Lf, and Hb levels. Each horizontal axis represents the endoscopic scores of MES, total UCEIS score for UC, and SES‐CD for CD.

We examined the correlation between fecal markers and both MES and UCEIS scores for UC and SES‐CD scores for CD. Fecal markers were significantly correlated with both MES (Cp, *P* < 0.001, *r*_*s*_ = 0.73; Lf, *P* < 0.001, *r*_*s*_ = 0.71; Hb, *P* < 0.001, *r*_*s*_ = 0.76) and total UCEIS (Cp, *P* = 0.01, *r*_*s*_ = 0.52; Lf, *P* = 0.015, *r*_*s*_ = 0.50; Hb, *P* < 0.01, *r*_*s*_ = 0.60). However, SES‐CD did not correlate with these fecal markers (Fig. 4).

Regarding disease activity based on the endoscopic severity of UC, fecal Cp, Lf, and Hb levels were significantly higher in patients with UC of MES 2–3 than in those with MES 0–1 (Cp, *P* = 0.07; Lf, *P* = 0.07; Hb, *P* = 0.03) (Fig. S3). In addition, fecal Cp, Lf, and Hb levels were significantly higher in patients with UC with MES 1–3 than in those with MES 0 (Cp, *P* < 0.01; Lf, *P* = 0.01; Hb, *P* < 0.01) (Fig. S4). By ROC analysis, the best discriminating cutoff levels for predicting MES ≥2 were fecal Cp of 984 μg/g, Lf of 713 μg/g, and Hb of 641 ng/mL (Fig. S3), and for predicting MES ≥1 were fecal Cp of 164 μg/g, Lf of 85 μg/g, and Hb of 38 ng/mL (Fig. S4). None of the fecal markers correlated with endoscopic scores for CD (data not shown).

### Correlation between fecal markers and serum inflammatory markers in IBD

We analyzed the correlation between fecal markers and serum inflammatory markers, such as C‐reactive protein (CRP). In patients with CD, fecal Hb moderately correlated with CRP (*P* = 0.028, *r*_*s*_ = 0.55) (Fig. S5).

### Relationship between fecal markers and colorectal tumor

#### Cutoff levels of fecal markers in the healthy control group

In this study, the mean values of Cp, Lf, and Hb in HCs were 127 μg/g (SD 74.9), 37 μg/g (SD 25.8), and 12 ng/mL (SD 9.1), respectively. With no general cutoff values of fecal Cp and Lf for colorectal tumors, we defined the upper cutoff values for each fecal marker as the mean + 2SD in the HC group; 270 μg/g for Cp and 85 μg/g for Lf.

#### Ability of fecal markers to detect advanced neoplasia and colorectal cancer

**Figure 5:**
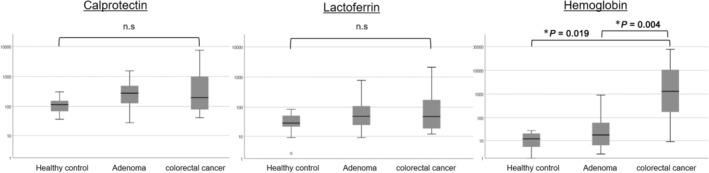
The difference in each fecal marker level in patients with colorectal tumors and in healthy controls. The levels of fecal markers in patients with colorectal cancer and adenoma and in HCs. The vertical axis in each figure is log‐transformed. Kruskal–Wallis test, followed by Tukey's multiple comparison tests, was applied to compare continuous variables with three groups.

We compared fecal marker levels in patients with colorectal cancer and adenoma with those in HCs. Fecal Hb levels were significantly higher in patients with colorectal cancer than those with adenoma (*P* = 0.004) and in HCs (*P* = 0.019) (Fig. 5). Although fecal Cp and Lf in patients with colorectal tumors tended to be higher than those in HCs, there were no significant differences in fecal Cp and Lf among the three groups (Fig. 5).

Next, we validated the ability of fecal Cp, Lf, and Hb levels, alone or in combination, to detect advanced neoplasia and colorectal cancer. Advanced adenoma was defined as any adenoma >10 mm or an adenoma with villous components more than 20% or with high‐grade dysplasia.^12^, ^13^ Based on the data from HCs, we set the upper cutoff values of fecal Cp and Lf as 270 and 85 μg/g, respectively. In addition, we defined the cutoff value for fecal Hb as 100 ng/mL, which is commonly adopted for colorectal cancer screening.

Fecal Hb alone had the best sensitivity (78.6%), specificity (85.1%), positive predictive value (PPV, 61.1%), and negative predictive value (NPV, 93%) for detecting colorectal cancer (*P* < 0.001); and the positive impact of adding fecal Cp and Lf was limited (Table 3). In the analysis to detect advanced adenoma plus colorectal cancer, fecal Hb had the best specificity (87.5%) and PPV (79.5%); however, adding Cp and Lf increased sensitivity and NPV. In contrast, the specificity was lower than that of Hb alone (Table 3).

**Table 3:** Diagnostic accuracy of fecal calprotectin, lactoferrin, and hemoglobin alone, and in combination

		Sensitivity	Specificity	PPV	NPV	*P* value
Advanced neoplasia	Cp ≧ 270 μg/g	71.4%	67.5%	27.8%	93.1%	0.064**
	Lf ≧ 85 μg/g	42.9%	80%	27.2%	88.9%	0.20**
	Hb ≧ 100 ng/mL	14.3%	87.5%	16.7%	85.4%	0.64**
	Cp ≧ 270 μg/g + Hb ≧ 100 ng/mL	83.3%	65.9%	27.8%	93.1%	0.64**
	Lf ≧ 85 μg/g + Hb ≧ 100 ng/mL	42.9%	72.5%	21.4%	87.9%	0.34**
	Cp ≧ 270 μg/g + Lf ≧ 85 μg/g	83.3%	65.9%	27.8%	93.1%	0.64**
	Cp ≧ 270 μg/g + Lf ≧ 85 μg/g + Hb ≧ 100 ng/mL	62.5%	61.5%	23.8%	92.3%	0.13**
Colorectal cancer	Cp ≧ 270 μg/g	42.9%	61.7%	25%	78.4%	0.76*
	Lf ≧ 85 μg/g	42.9%	75%	35.3%	81.8%	0.15*
	Hb ≧ 100 ng/mL	78.6%	85.1%	61.1%	93%	**<0.001** **
	Cp ≧ 270 μg/g + Hb ≧ 100 ng/mL	57.1%	61.7%	30.8%	82.9%	0.17*
	Lf ≧ 85 μg/g + Hb ≧ 100 ng/mL	78.6%	70.2%	44%	91.7%	**0.016** **
	Cp ≧ 270 μg/g + Lf ≧ 85 μg/g	57.1%	57.4%	28.6%	81.8%	0.34*
	Cp ≧ 270 μg/g + Lf ≧ 85 μg/g + Hb ≧ 100 ng/mL	78.6%	55.3%	34.4%	89.7%	**0.026** **
Advanced neoplasia + colorectal cancer	Cp ≧ 270 μg/g	52.4%	67.5%	45.8%	73.0%	0.16*
	Lf ≧ 85 μg/g	42.9%	80%	52.9%	72.7%	0.059*
	Hb ≧ 100 ng/mL	57.1%	87.5%	70.6%	79.5%	**<0.001** *
	Cp ≧ 270 μg/g + Hb ≧ 100 ng/mL	61.9%	67.5%	50%	77.1%	**0.027** *
	Lf ≧ 85 μg/g + Hb ≧ 100 ng/mL	66.7%	72.5%	56%	80.6%	**0.003** *
	Cp ≧ 270 μg/g + Lf ≧ 85 μg/g	57.1%	67.5%	48%	77.1%	0.057*
	Cp ≧ 270 μg/g + Lf ≧ 85 μg/g + Hb ≧ 100 ng/mL	76.2%	60%	50%	82.8%	**0.007** *

* *χ* ^2^ test.

** Fisher's exact test.

Abbreviation: Cp, Calprotectin; Lf, Lactoferrin; Hb, Hemoglobin; PPV, Positive predictive value; NPV, Negative predictive value.

#### Relationship between fecal markers and tumor size and invasion depth in colorectal cancer

The median maximum diameter of colorectal tumors, excluding carcinoma, was 6.5 mm (range, 2–18 mm) (Table S1). Although we examined the association between polyp size and fecal markers, no significant correlation was observed (Fig. S6). In colorectal cancer, fecal markers were not significantly different according to lesion location (Fig. S7); however, fecal Cp and Lf tended to be higher in patients with T4 colorectal cancer, as defined by the TNM classification system, than in those with T1 to T3 cancer (Fig. S7).

## Discussion

This is the first clinical validation study to measure fecal Cp, Lf, and Hb levels using an all‐in‐one kit in patients with IBD and colorectal tumors. In this study, we found that fecal Cp and Lf are almost equivalent in assessing clinical activity in patients with UC and that Cp and Lf are elevated in colorectal tumors, especially colorectal cancer.

Regarding IBD, our data showed that fecal Cp and Lf levels were significantly higher in patients with clinically active UC and were correlated with both partial Mayo and endoscopic scores. The results of this study showed that the three fecal markers significantly correlated with MES and had higher accuracy in distinguishing MES 0 from MES ≥1 rather than MES 0–1 from MES ≥2.

Fecal biomarkers are expected to be a noninvasive alternative to colonoscopy for the management of IBD.^14^ Among the neutrophil‐derived proteins in the feces, Cp, Lf, and neutrophil elastase are sensitive markers for evaluating the disease activity of IBD and differentiating it from irritable bowel syndrome.^15^ Several reports have demonstrated that fecal Cp is a widely accepted noninvasive marker for assessing the clinical and endoscopic activities of patients with IBD.^1^ In this study, we examined fecal Lf and Hb levels to assess disease activity in patients with IBD.

Based on the results of the ROC analysis, fecal Cp and Lf had almost equivalent abilities to detect clinical remission in patients with UC; however, fecal Cp was slightly superior to Lf. Because Lf exerts its antimicrobial activity through an iron‐dependent mechanism, it may not have adequately reflected the disease state of UC patients with chronic anemia.^16^ In this study, fecal Cp and Lf did not correlate with disease activity in patients with CD. Zittan et al. suggested that fecal Cp does not correlate well with disease activity in small intestinal CD because Cp is degraded by proteases normally present in the small intestine.^17^ Generally, patients with CD who had small bowel involvement often lack clinical symptoms; therefore, we could not find an association between fecal biomarkers and clinical activity. Fecal Cp and Lf are more suitable for evaluating inflammation in the colon than in the small intestine, and we should know the disease type in CD when using fecal biomarkers.

Fecal immunoassay test (FIT) is a standard screening method for colorectal cancer detection. Despite a high specificity of 88–97.7%, its sensitivity is insufficient for screening colorectal cancer.^18^, ^19^, ^20^, ^21^, ^22^, ^23^, ^24^ Testing FIT on two consecutive days is recommended to compensate for the low sensitivity because FIT may produce false‐negatives due to improper stool storage and decreased sensitivity due to the degradation of Hb during prolonged retention in the intestine.^25^, ^26^ Fecal Cp and Lf levels tended to be higher in patients with adenomas and colorectal cancer than in HCs, as reported previously.^27^, ^28^ Fecal Hb had a sensitivity of 78.6% and specificity of 85.1% for detecting colorectal cancer, which was superior to other fecal markers alone or in combination. Although fecal inflammatory markers were elevated in patients with colorectal tumors, including benign and malignant tumors, fecal Cp and Lf did not have as much utility as fecal Hb in detecting advanced neoplasm and colorectal cancer in this study. However, fecal Hb alone has relatively low sensitivity for detecting colorectal tumors, and adding Cp or Lf may reduce false‐negatives. As chronic inflammation is involved in the development of colorectal tumors, these inflammatory markers in the intestinal tract may be suitable for detecting tumors at an early stage.^29^ The number and size of colorectal tumors did not correlate with any fecal markers in this study. Based on an analysis of patients with colonic polyposis, fecal Cp levels were not significantly different according to the number and macroscopic features.^30^ Previous reports suggested that higher levels of fecal Cp were positively correlated with the T‐stage of colorectal cancer based on the seventh edition of the TNM classification.^27^, ^31^ Similarly, our data showed that fecal Cp and Lf tended to be higher in patients with T4 cancers than in those with T1 cancers, indicating that elevated fecal Cp and Lf might be associated with the progression of colorectal cancer.

In conclusion, fecal Cp and Lf levels can be used as equivalent biomarkers to assess the clinical activity in patients with UC. Fecal Hb is the most useful marker for screening colorectal cancer; however, adding fecal Cp and Lf may compensate for the low sensitivity of detecting for advanced colorectal cancer based on Hb alone.

## Limitation

First, we did not exclude patients taking oral antithrombotic medications, which may have affected fecal Hb levels. Second, although we did not perform risk stratification according to comorbidities, patients with systemic inflammation that could affect fecal markers were excluded. Third, we could not calculate the number of patients needed for analysis because the present study was exploratory analysis with three fecal biomarkers.

## Data availability statement

The data that support the findings of this study are available from the corresponding author on reasonable request due to privacy or ethical restrictions.

## Ethics approval statement

This study was approved by the ethics committee of Sapporo Medical University (IRB No. 312–132) and all procedures were performed in accordance with the ethical standards of the 1964 Declaration of Helsinki and its later amendments.

## Patient consent statement

Written informed consent was obtained from all the enrolled patients.

## Supporting information


**Figure S1.** The difference in fecal markers in each group. The levels of (A) fecal Cp, (B) Lf, and (C) Hb in patients with colorectal carcinoma, adenoma, and healthy controls. A value of *P* < 0.05 was statistically significant, and the Kruskal–Wallis test followed by Tukey's multiple comparison tests was applied. The values of the vertical axis are log‐transformed.

**Figure S2.** The difference in fecal markers in patients with clinically active CD and those in remission. The difference in fecal marker levels in patients with clinically active CD (simple CDAI >4) and those in remission. (A) calprotectin, (B) lactoferrin, and (C) hemoglobin. The value on vertical axis is log‐transformed. ROC analysis of (D) fecal Cp and (E) Lf for detecting clinically active CD.

**Figure S3.** The difference in fecal markers between patients with UC with MES 0–1 and MES 2–3. Differences in fecal marker levels between patients with UC with MES 0–1 and MES 2–3. (A) calprotectin, (B) lactoferrin, and (C) hemoglobin. The value on the vertical axis is log‐transformed. ROC analysis of (D) fecal Cp and (E) Lf to detect MES >1.

**Figure S4.** The difference in fecal markers between patients with UC with MES 0 and MES 1–3. The difference in fecal marker levels between patients with UC with MES 0 and MES 1–3. (A) calprotectin, (B) lactoferrin, and (C) hemoglobin. The value on the vertical axis is log‐transformed. ROC analysis of (D) fecal Cp and (E) Lf for detecting MES >0.

**Figure S5.** The relationship between fecal markers and CRP in patients with IBD. Association between fecal markers and CRP levels in patients with UC (A–C) and CD (D–F). The vertical axes represent the fecal Cp, Lf, and Hb levels, and the horizontal axes represent CRP.

**Figure S6.** Fecal markers by polyp size in patients with colorectal tumors. Correlation between fecal markers ((A) calprotectin, (B) lactoferrin, and (C) hemoglobin) and polyp size (mm).

**Figure S7.** Fecal markers by the location and invasion depth in patients with invasive colorectal cancers. Differences in fecal marker levels by location ((A) calprotectin, (B) lactoferrin, and (C) hemoglobin) and T‐stage ((D) calprotectin, (E) lactoferrin, and (F) hemoglobin) in patients with invasive colorectal cancers.

**Table S1.** Characteristic of patients with colorectal tumor.

**Table S2.** Characteristics of patients with IBD.
