# Supplementary figures and images for: Clinical performance of fecal calprotectin, lactoferrin, and hemoglobin for evaluating the disease activity of IBD and detecting colorectal tumors
Source: JGH Open. 2024 Jun 4;8(6):e13077. doi: 10.1002/jgh3.13077 (PMC11148478; doi:10.1002/jgh3.13077)

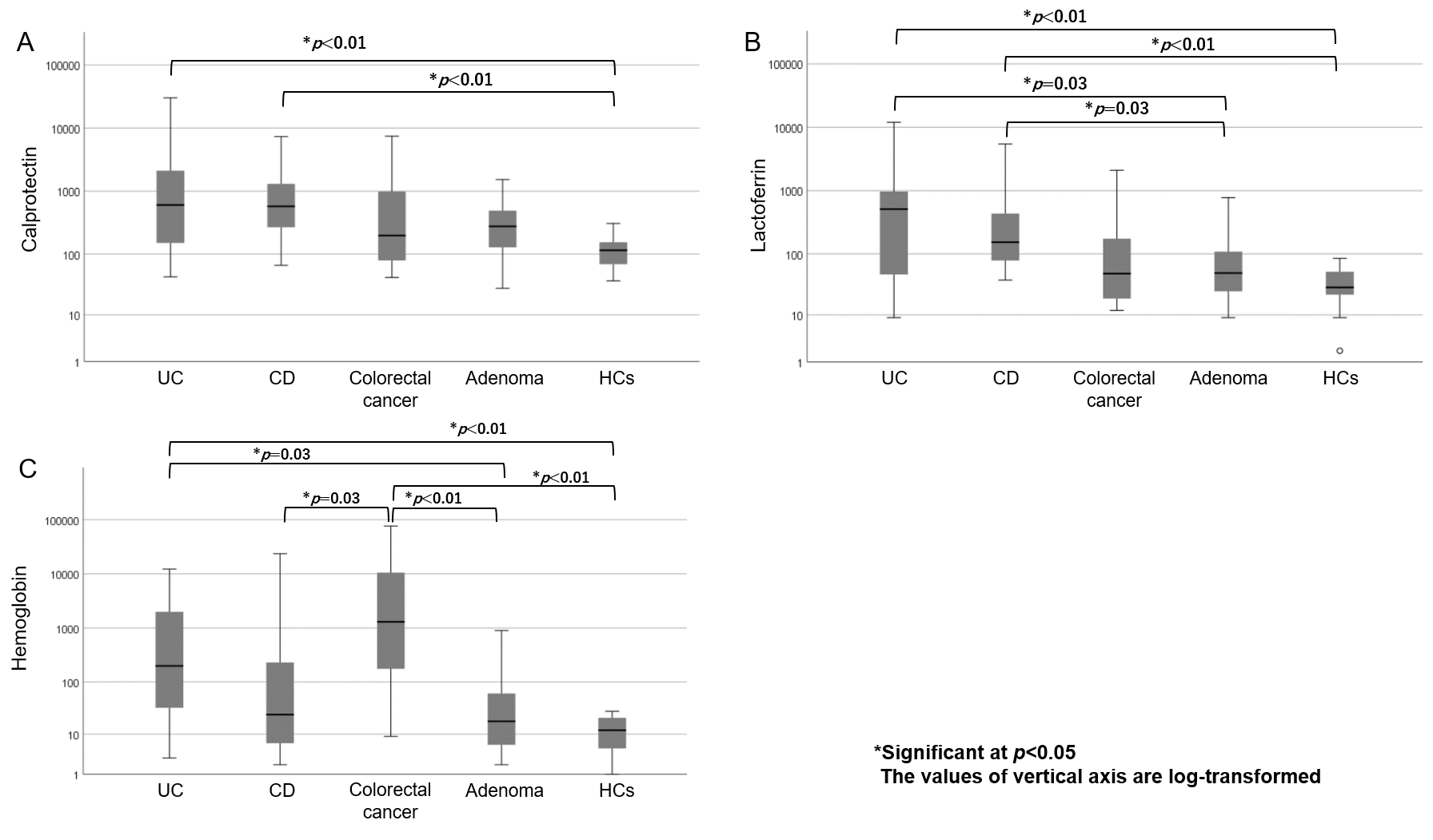

Supplement: Supplementary file 1 — Figure S1. The difference in fecal markers in each group. The levels of (A) fecal Cp, (B) Lf, and (C) Hb in patients with colorectal carcinoma, adenoma, and healthy controls. A value of P < 0.05 was statistically significant, and the Kruskal–Wallis test followed by Tukey's multiple comparison tests was applied. The values of the vertical axis are log‐transformed. [file JGH3-8-e13077-s002.tif]

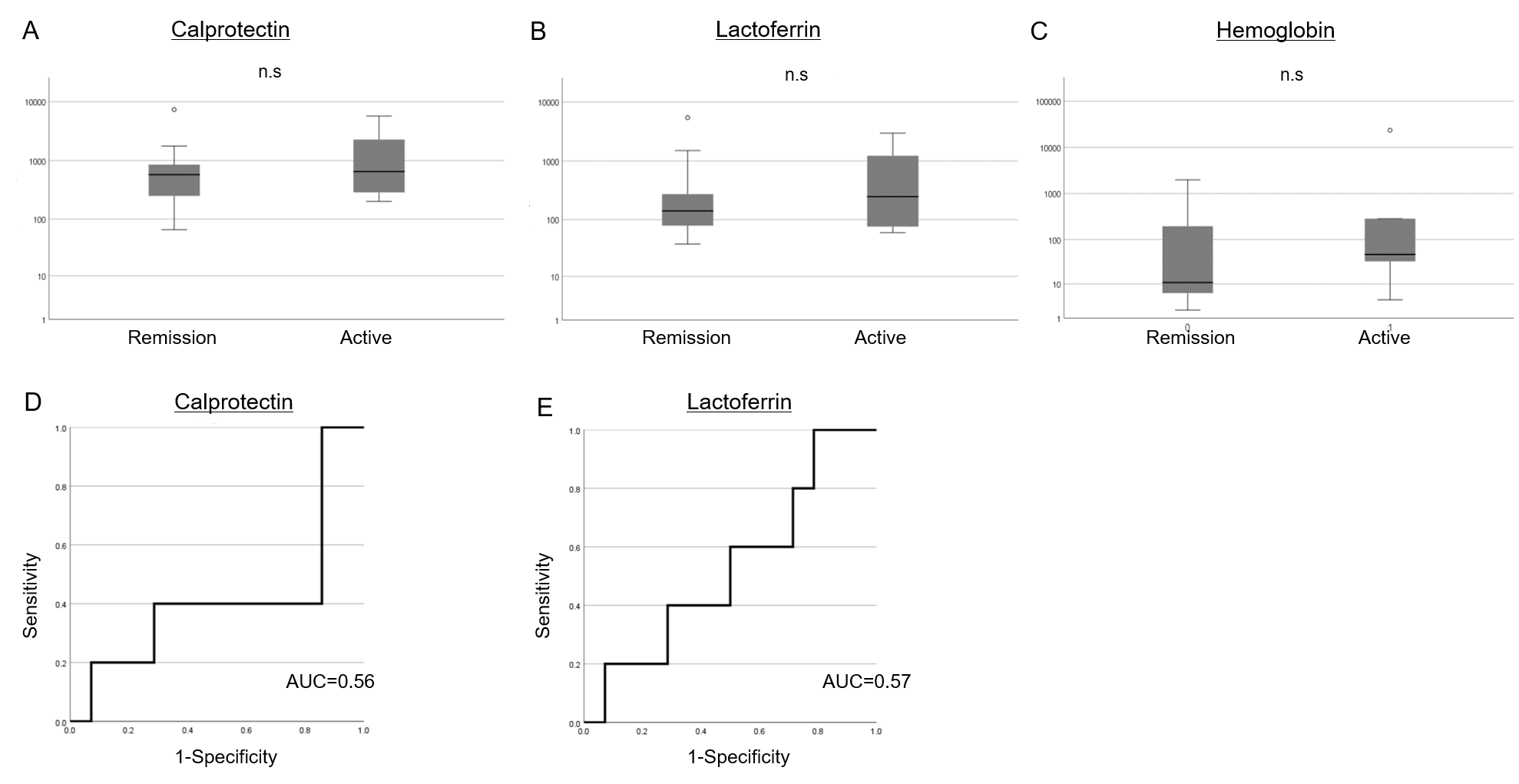

Supplement: Supplementary file 2 — Figure S2. The difference in fecal markers in patients with clinically active CD and those in remission. The difference in fecal marker levels in patients with clinically active CD (simple CDAI >4) and those in remission. (A) calprotectin, (B) lactoferrin, and (C) hemoglobin. The value on vertical axis is log‐transformed. ROC analysis of (D) fecal Cp and (E) Lf for detecting clinically active CD. [file JGH3-8-e13077-s003.tif]

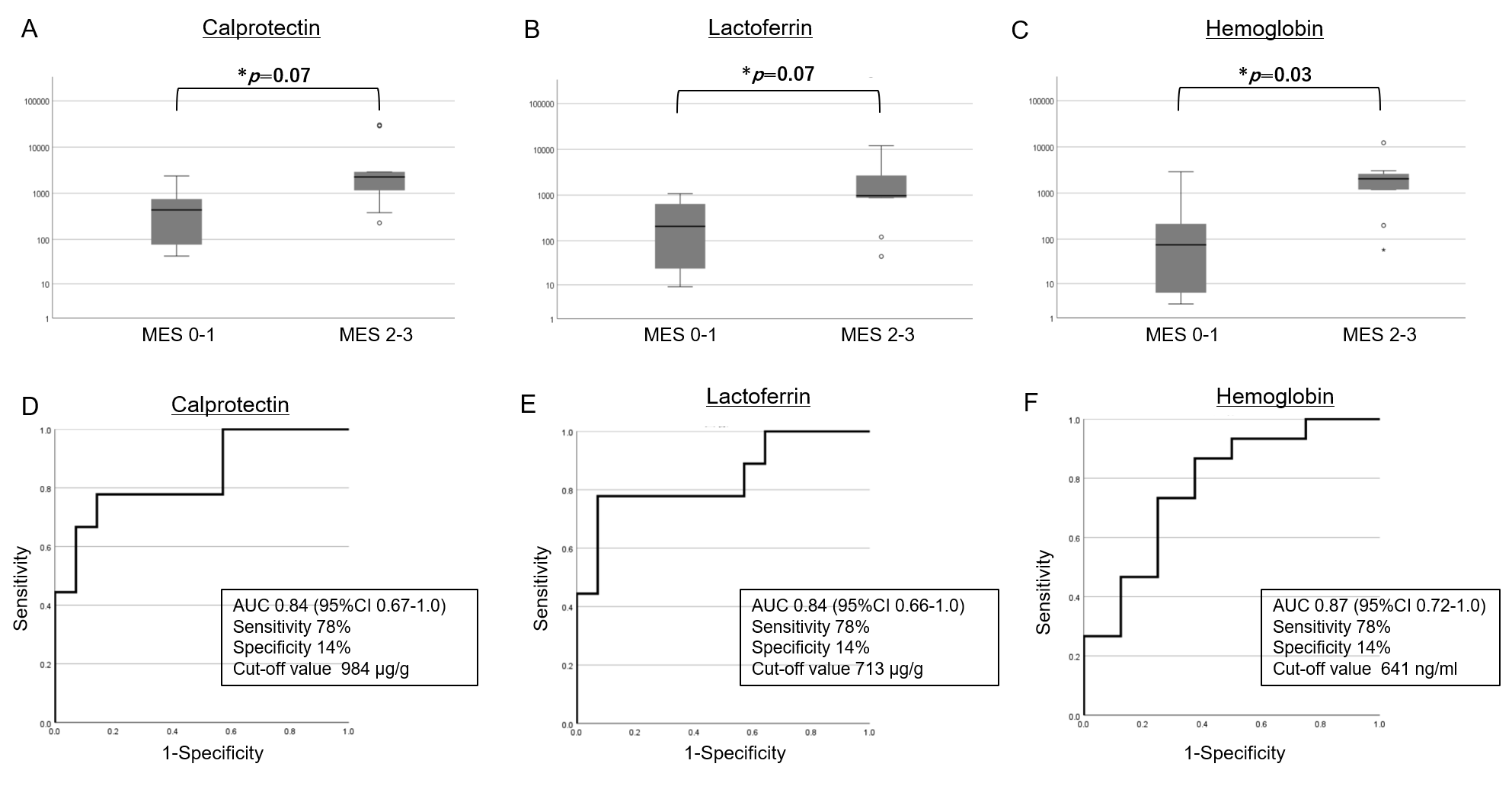

Supplement: Supplementary file 3 — Figure S3. The difference in fecal markers between patients with UC with MES 0–1 and MES 2–3. Differences in fecal marker levels between patients with UC with MES 0–1 and MES 2–3. (A) calprotectin, (B) lactoferrin, and (C) hemoglobin. The value on the vertical axis is log‐transformed. ROC analysis of (D) fecal Cp and (E) Lf to detect MES >1. [file JGH3-8-e13077-s001.tif]

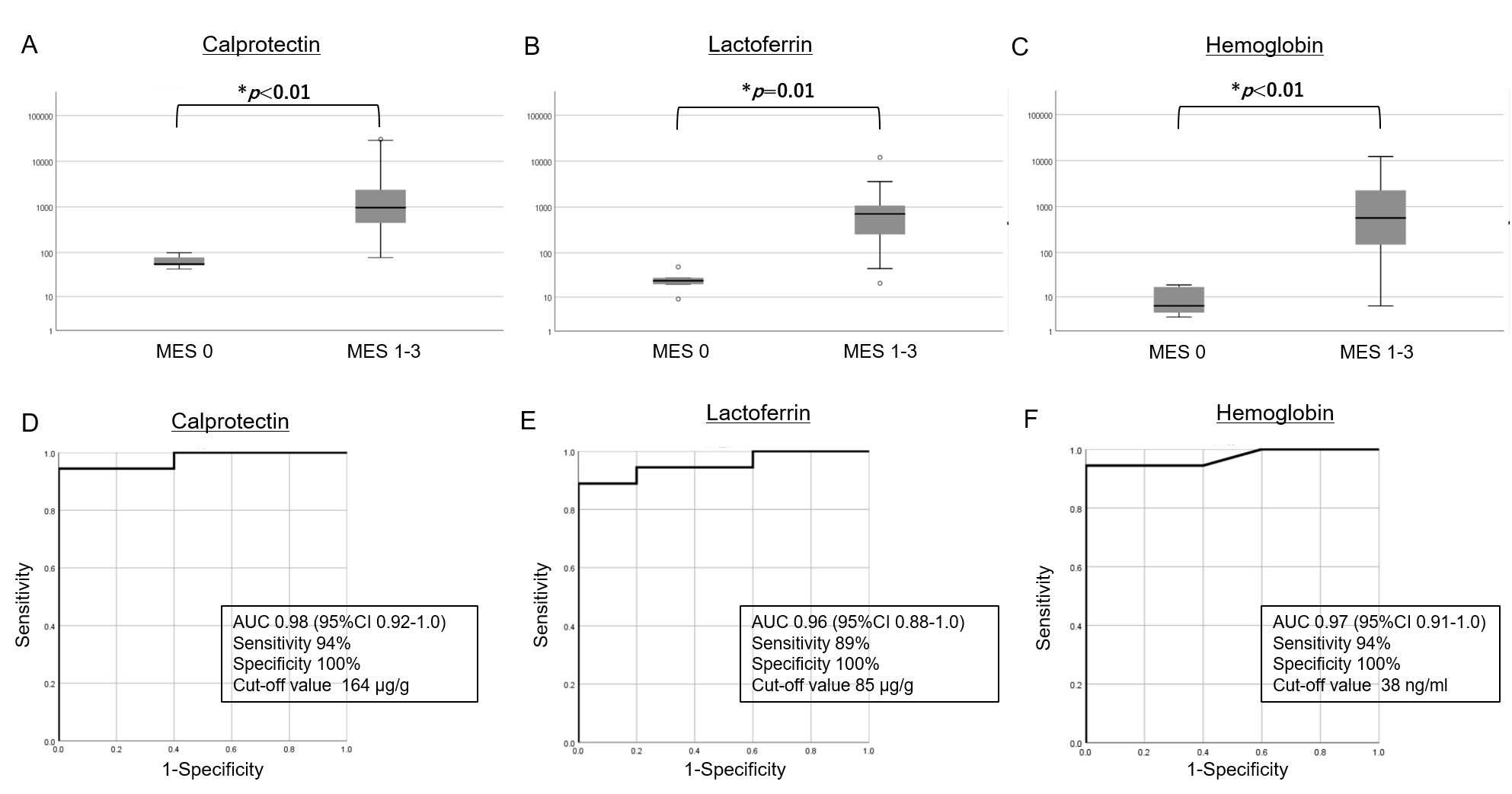

Supplement: Supplementary file 4 — Figure S4. The difference in fecal markers between patients with UC with MES 0 and MES 1–3. The difference in fecal marker levels between patients with UC with MES 0 and MES 1–3. (A) calprotectin, (B) lactoferrin, and (C) hemoglobin. The value on the vertical axis is log‐transformed. ROC analysis of (D) fecal Cp and (E) Lf for detecting MES >0. [file JGH3-8-e13077-s008.tif]

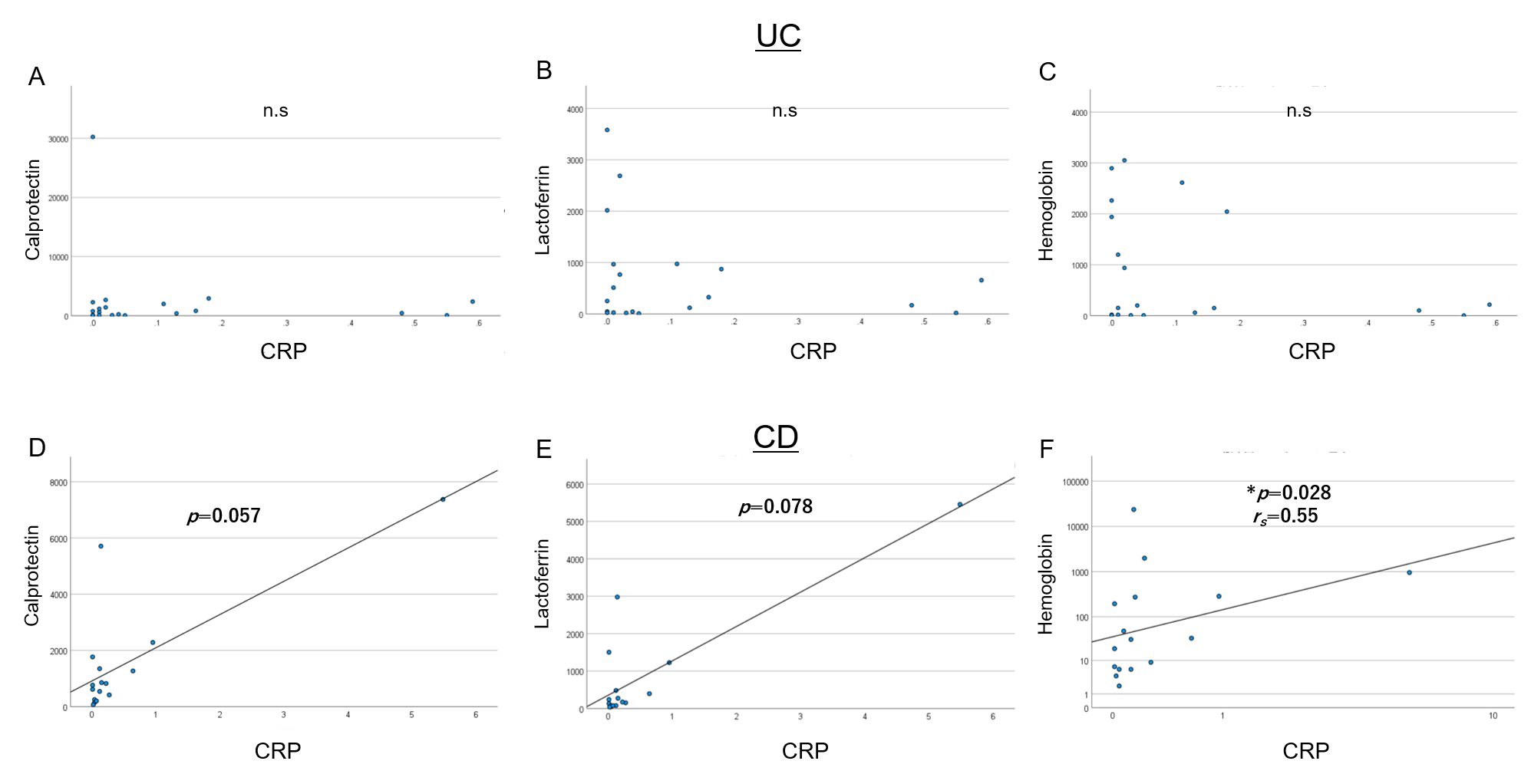

Supplement: Supplementary file 5 — Figure S5. The relationship between fecal markers and CRP in patients with IBD. Association between fecal markers and CRP levels in patients with UC (A–C) and CD (D–F). The vertical axes represent the fecal Cp, Lf, and Hb levels, and the horizontal axes represent CRP. [file JGH3-8-e13077-s004.tif]

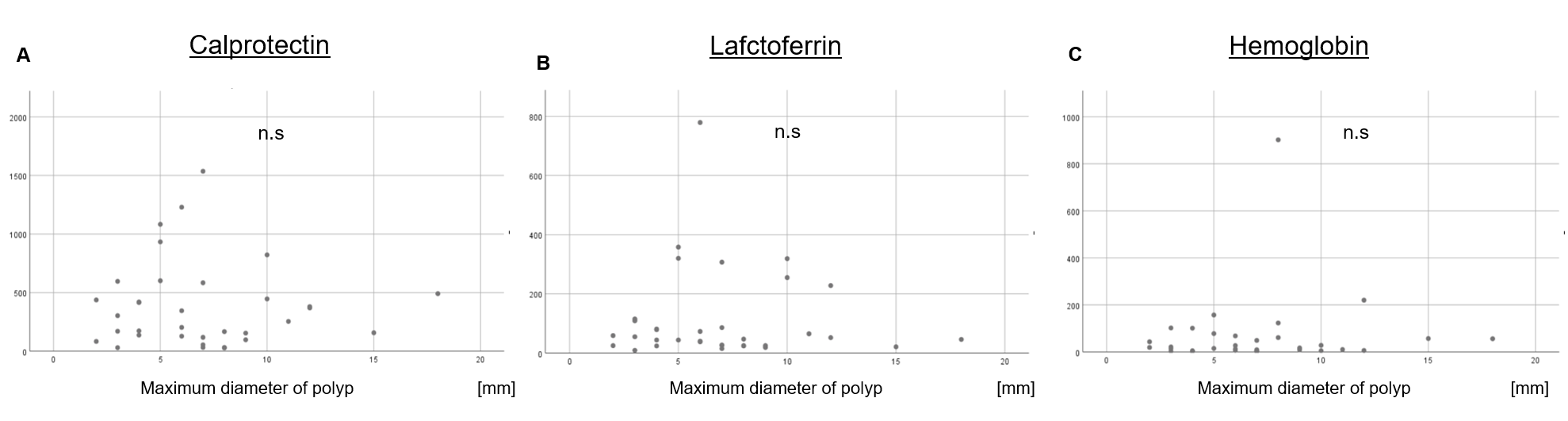

Supplement: Supplementary file 6 — Figure S6. Fecal markers by polyp size in patients with colorectal tumors. Correlation between fecal markers ((A) calprotectin, (B) lactoferrin, and (C) hemoglobin) and polyp size (mm). [file JGH3-8-e13077-s005.tif]

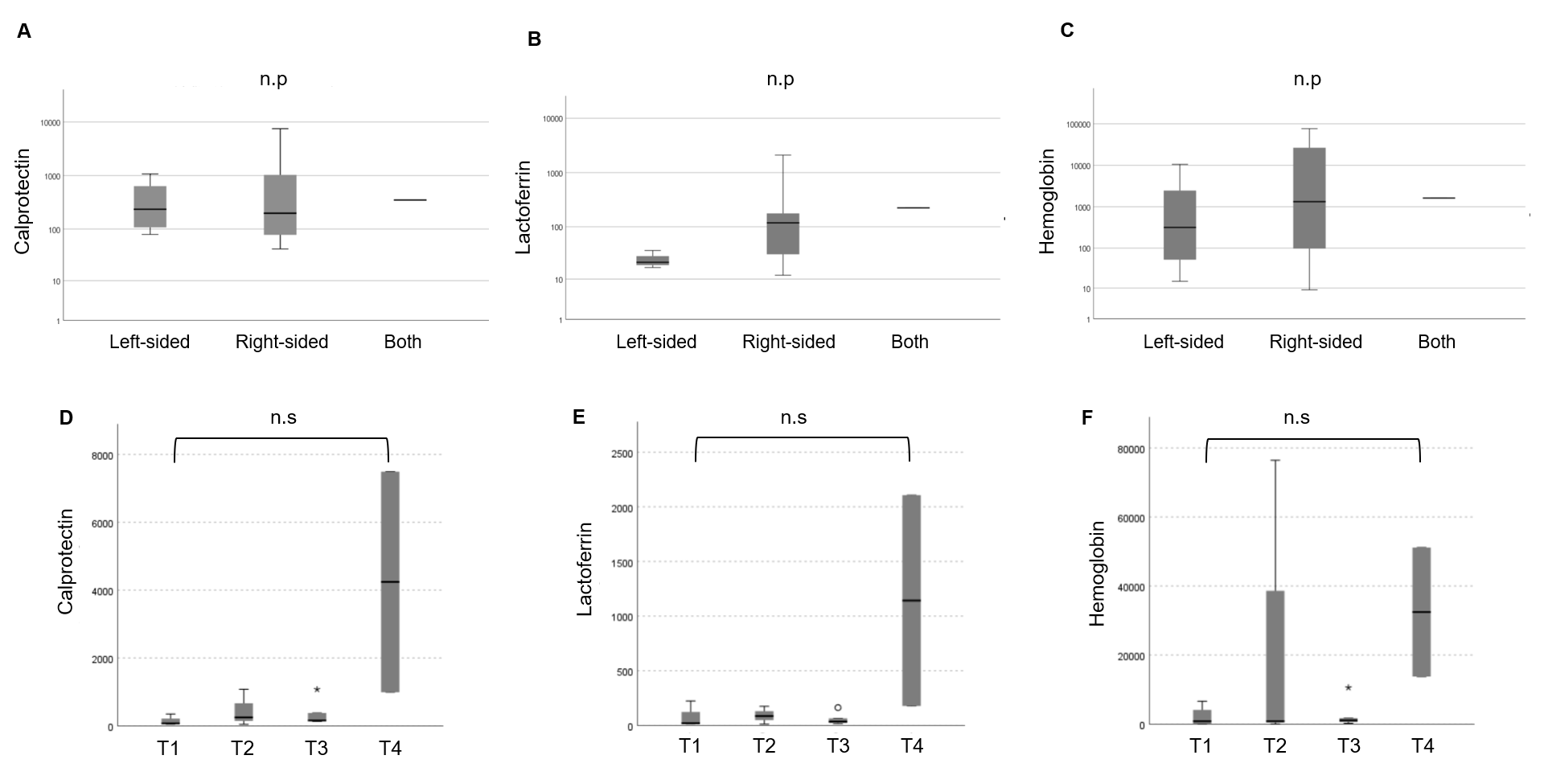

Supplement: Supplementary file 7 — Figure S7. Fecal markers by the location and invasion depth in patients with invasive colorectal cancers. Differences in fecal marker levels by location ((A) calprotectin, (B) lactoferrin, and (C) hemoglobin) and T‐stage ((D) calprotectin, (E) lactoferrin, and (F) hemoglobin) in patients with invasive colorectal cancers. [file JGH3-8-e13077-s006.tif]

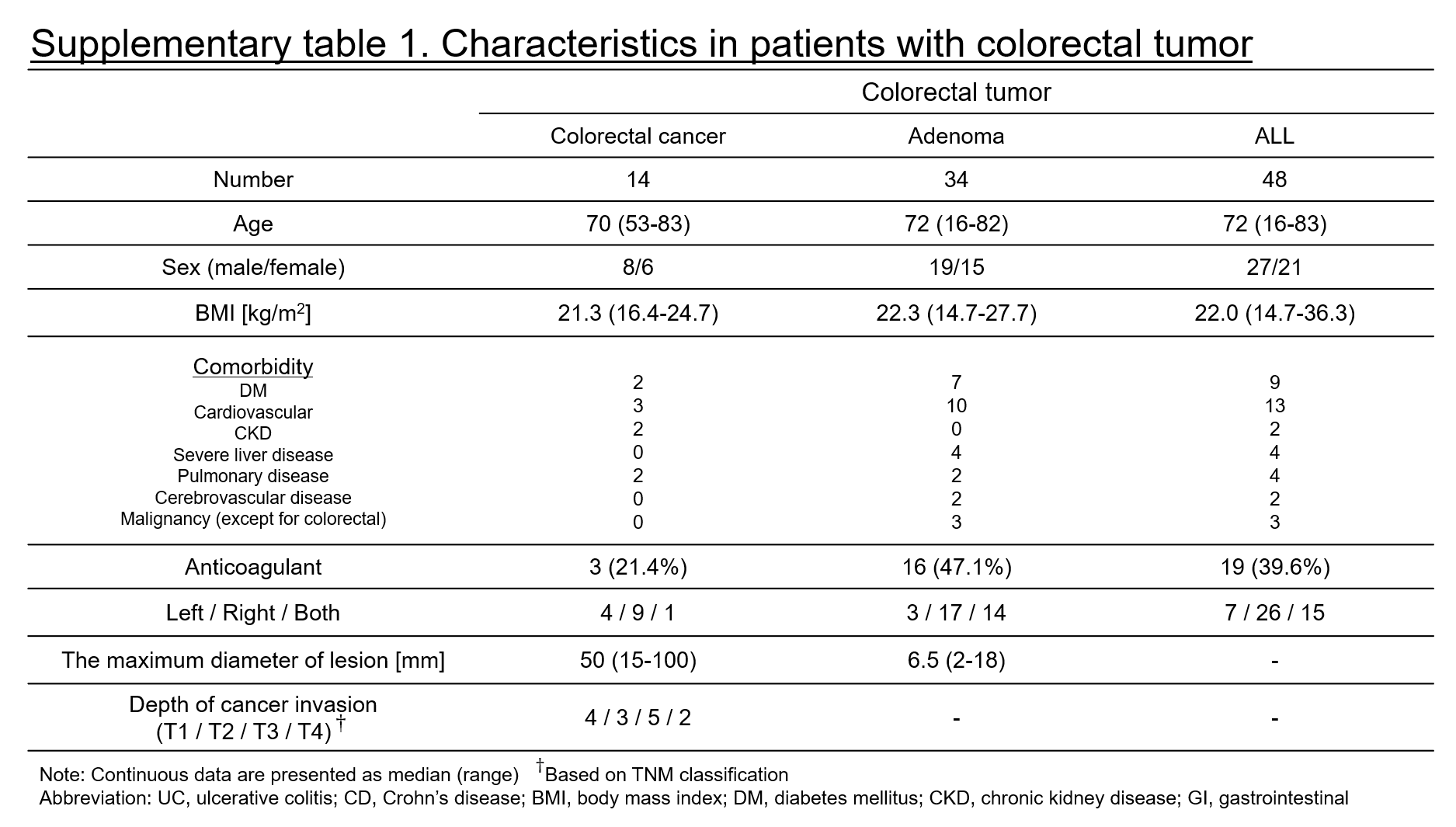

Supplement: Supplementary file 8 — Table S1. Characteristic of patients with colorectal tumor. [file JGH3-8-e13077-s009.tif]

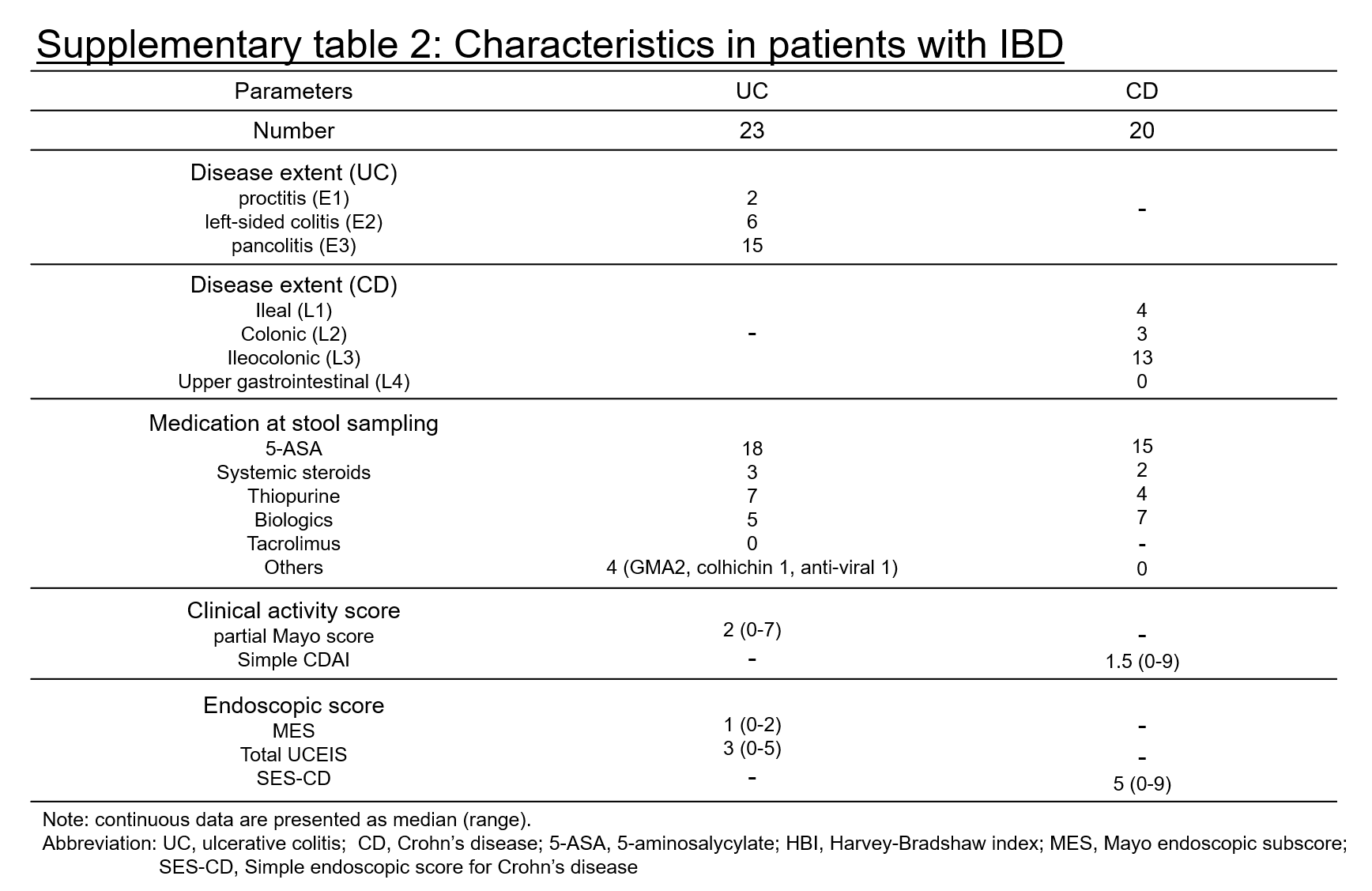

Supplement: Supplementary file 9 — Table S2. Characteristics of patients with IBD. [file JGH3-8-e13077-s007.tif]
